# Interactions of VEGF 1154A and 2578C with APOE ε4 on Amyloid Load in Cognitively Unimpaired Older Adults

**DOI:** 10.1002/alz.086589

**Published:** 2025-01-09

**Authors:** Michael H. Malek‐Ahmadi, Ignazio Piras, Qi Wang, Kewei Chen, Vivek Devadas, Ji Luo, Yi Su

**Affiliations:** ^1^ Banner Alzheimer's Institute, Phoenix, AZ USA; ^2^ Arizona Alzheimer's Consortium, Phoenix, AZ USA; ^3^ University of Arizona College of Medicine‐Phoenix, Phoenix, AZ USA; ^4^ The Translational Genomics Research Institute (TGen‐ an Affiliate of City of Hope), Phoenix, AZ USA; ^5^ Arizona State University, Tempe, AZ USA; ^6^ ASU‐Banner Neurodegenerative Disease Research Center, Tempe, AZ USA; ^7^ School of Mathematics and Statistical Sciences, College of Health Solutions, Arizona State University, Tempe, AZ USA; ^8^ University of Arizona College of Medicine Phoenix, Phoenix, AZ USA; ^9^ ASU‐Mayo Center for Innovative Imaging, Tempe, AZ USA

## Abstract

**Background:**

Previous studies have shown that carriage of the VEGF 1154A (rs1570360) and the VEGF 2578C (rs699947) alleles may confer a protective effect on the development of Alzheimer’s disease (AD). However, it is unknown if these associations are APOE‐dependent and whether they can be observed in asymptomatic individuals with varying levels of amyloid pathology. The aim of this study is to determine whether interactions between the APOE ε4 allele, VEGF 1154A, and VEGF 2578C are associated with amyloid load in cognitively unimpaired (CU) older adults.

**Method:**

Data from 341 CU ADNI subjects (57% female) with a mean age of 74.35±6.94 years and a mean education level of 16.63±2.44 years were included in the analysis. Thirty‐two percent (n=109) were APOE ε4 carriers with the following distributions for each VEGF allele: 1154A – GG=165, AG=147, AA=29; 2578C – AA=88, AC=166, CC=87. AV‐45 mean cortical standard uptake value ratio (MCSUVR) was used to determine amyloid load. Generalized linear models were used to quantify the main effects of the VEGF alleles and their interactions with APOE ε4 on demographically‐adjusted MCSUVR values. Cohen’s d effect size was used for groupwise comparisons.

**Result:**

The interaction between VEGF 1154A and APOE was statistically significant (β = ‐0.02, 95% CI (‐0.04, ‐0.008), p = 0.002). Further analysis among those with two A alleles of VEGF 1154 found that APOE ε4 carriers had substantially lower amyloid load relative to ε4 non‐carriers (p = 0.01, d = 1.04). Within APOE ε4 carriers, there were no significant differences among the GG, AG, and AA genotypes. The interaction for VEGF 2578C and APOE approached statistical significance for amyloid load (β = 0.01, 95% CI (‐0.001, 0.03), p = 0.06).

**Conclusion:**

The AA genotype of VEGF 1154 is associated with reduced amyloid load in APOE ε4 carriers and warrants further investigation as a potential protective factor for AD.

